# Predicting invasion risk of grasses in novel environments requires improved genomic understanding of adaptive potential

**DOI:** 10.1002/ajb2.16079

**Published:** 2022-11-28

**Authors:** Emily S. Bellis, Rima D. Lucardi, Kristin Saltonstall, Travis D. Marsico

**Affiliations:** ^1^ Department of Computer Science, Arkansas State University State University AR USA; ^2^ Center for No‐Boundary Thinking, Arkansas State University State University AR USA; ^3^ Southern Research Station United States Department of Agriculture Forest Service Athens GA USA; ^4^ Smithsonian Tropical Research Institute Balboa Ancon Panamá; ^5^ Department of Biological Sciences, Arkansas State University State University AR USA

**Figure 1:**
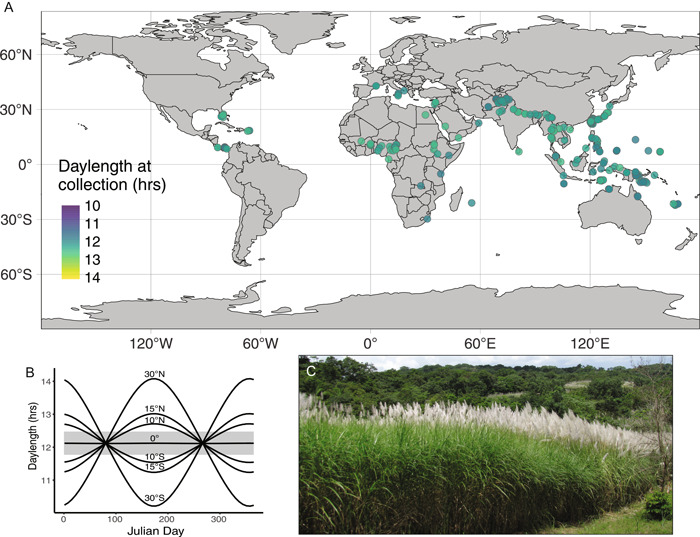
(A) Distribution of 330 *Saccharum spontaneum* herbarium accessions from the Global Biodiversity Information Facility (GBIF.org, 2022). Despite a relatively broad geographic distribution of collected specimens, hours of daylight on the day of collection represents a narrow range (11.8–12.5 h). Collection date is considered here as a proxy for flowering time, since most collectors attempt to preserve specimens in reproductive phases. (B) Photoperiod across the year at a subset of latitudes where *S. spontaneum* occurs (0–30°). Horizontal gray shading delineates the range of values for daylength during the collection of the 330 georeferenced *S. spontaneum* herbarium accessions. Without gene flow from other species, introduced populations of wild sugarcane at locations far from the equator may have a more restricted window for flowering or may not flower at all if daylength changes are too rapid around the autumnal equinox. (C) Invasive *S. spontaneum* bordering tropical forest in Soberanía National Park, Panamá.

Many invasive grasses drastically transform the ecosystems they invade, putting native communities across the globe at risk (Fusco et al., 2019). Climate change will likely increase invasion risks for many regions as climates in temperate zones become warmer (Bellard et al., 2012) and tropical and semi‐tropical species are able to expand their distributions. However, other axes of environmental variation could delay invasions. For example, invaders from low‐latitude‐adapted source populations may not flower at higher latitudes because of differences in photoperiod sensitivity (Brandes, 1950; Box 1). Though many nonnative and invasive grasses can quickly establish and persist asexually by rhizomatous reproduction and local spread, their ability to flourish and become widespread in new locations may depend on sexual reproduction and influx of novel genetic variation from other populations or species. Gene flow among introduced lineages and subsequent selection in new environments may have facilitated success of several established perennial grass invaders including *Bromus tectorum* (Novak and Mack, 1993) and *Imperata cylindrica* (Lucardi et al., 2014) throughout the United States and *Saccharum spontaneum* (Saltonstall et al., 2021) in Panama (Figure 1). These species have all had incredibly damaging impacts by altering fire regimes and displacing native communities in their invaded ranges (Hooper et al., 2005; Fusco et al., 2019). With inherent challenges when predicting the fitness of an organism in new environments, incorporating adaptive potential into predictions of invasion risk remains a challenging frontier in an era of global change (Prentis et al., 2008).

Box 1 The USDA‐listed Federal Noxious Weed *Saccharum spontaneum* (wild sugarcane) is an urgent and challenging case study for improved modeling of invasion risk, and a high‐quality genome and population genomic resequencing data are already available (Zhang et al., 2022). It has been reported as a weed in 33 countries and has high tolerance to biotic and abiotic stresses, including drought resistance and low nutrients. An intermediate‐day plant, *S. spontaneum* requires ~12 h of daylight and slowly decreasing daylength to flower (Figure 1), which may present a barrier to invasion at higher latitudes by limiting sexual reproduction and seed dispersal. Genomic study of adaptation in wild sugarcane populations is further complicated by its large genome (>10 Gb) and high, variable ploidy levels (2*n* = 40–128). In the United States, wild sugarcane is currently known to have naturalized only in Florida and Hawaii and has not yet become widespread despite its introduction for sugarcane breeding over 100 years ago (Figure 1). However, its propagules are entering the United States in large numbers and were documented as the most abundant taxon among seeds attached to the air‐intake grilles of refrigerated shipping cargo‐containers arriving at the Port of Savannah, Georgia (Lucardi et al., 2020). Hitchhiking seeds entering the United States through global trade routes represent a subset of global genetic diversity, suitable for fine‐scale genomic prediction of fitness at potential introduction sites and evaluating if (or when) *S. spontaneum* will become invasive in the United States.

The grasses (family Poaceae) are an exceptional model group for understanding rapid adaptive potential in the face of novel combinations of environmental parameters. Grasses were invading novel habitats even before the success of notorious lineages of the Anthropocene and have shown a remarkable ability to adapt to novel climatic conditions. C_4_ photosynthesis, for example, evolved multiple times independently in the grasses as diverse lineages adapted to seasonally dry, tropical environments (Linder et al., 2018). Several distinctive genomic features could contribute to the extraordinary ability of grasses to colonize, adapt to, and transform new environments. Polyploidy, for example, is common in grasses and could reduce the impacts of inbreeding in newly establishing populations or contribute to increased ecological plasticity (Linder et al., 2018). Porous species boundaries and high genomic tolerance for interspecific and intergeneric hybridization could further enhance adaptive responses, providing access to novel genetic material from species already well‐adapted to environments in invaded ranges. Moreover, grass genomes have high levels of heterozygosity and are dominated by repetitive sequences, which could be associated with increased trait variation because of expression plasticity among individuals (Stapley et al., 2015). Because of their importance to the global food supply, extensive genomic and germplasm resources are publicly available and continually being developed for cereal crops and their wild relatives, facilitating genome‐scale investigations.

As precipitous anthropogenic change continues to provide new opportunities for biological invasions, studies in grass systems will contribute to rapid progress on several long‐standing questions in invasion biology, including the following:

1. How important is adaptation for facilitating invasions in novel environments? Established populations often have a remarkable ability to succeed in new environments, but the extent to which this success is attributable to adaptation rather than to other processes such as plasticity is not always clear. When adaptation is important for invasion, to which axes of environment (e.g., photoperiod, drought, heat, biotic pressures) are populations able to adapt most quickly, and what is the underlying genetic architecture?
2. How frequent is adaptive introgression from genetically distinct lineages or species? To gauge the limit and rate of adaptive response within species, it is important to determine how easily environmentally adaptive alleles can be contributed by other taxa. An important first step is to answer the question “How accurately can adaptive introgression from closely related species be recognized?”
3. What genomic characteristics facilitate rapid adaptation? Invasive lineages may be more likely to have smaller genomes due to energetic costs of replication (Pyšek et al., 2018), though larger genomes may be associated with increased flexibility (Stapley et al., 2015). How do trade‐offs among invasiveness and genome size or complexity depend on environmental context at the introduction site (e.g., availability of nutrients required to replicate larger genomes) (Pyšek et al., 2018)?

With continued improvements in short‐ and long‐read sequencing technology, we are increasingly equipped to interrogate the genomes of nonmodel taxa and explore these questions. Parallel development of analytical approaches for integrating genomic and environmental data further lay the groundwork for a new era of genome‐informed invasion risk models. One promising framework, arguably not yet widely adopted for the purposes of invasion risk modeling, is that of genomic offset, a measure of the genetic distance between the actual (or predicted) genetic composition of a population and the set of alleles that may optimize fitness in a particular environment (Fitzpatrick and Keller, 2015). In practice, genomic offset has primarily been used to predict maladaptation for populations of conservation concern, fitting allele–environment relationships using a space‐for‐time substitution before projecting to the future to estimate the magnitude of genetic change required to keep up with a changing climate. In addition to validation studies with experiments (Fitzpatrick et al., 2021), recent developments in forward genetic simulation tools are enabling rigorous evaluation of supervised machine learning algorithms such as gradient forest for estimation of genomic offset (Láruson et al., 2022). Predicting invasion risk in response to both climate factors and photoperiod (Box 1) is a promising application of gradient forest, given its capacity to capture nonlinearity in relationships between genomic variants and multivariate environments.

While the genomic offset concept may facilitate improved prediction of invasion success across environments, *understanding* the genetic basis of adaptation to these environments will require complementary approaches. One powerful framework, genotype–environment association analysis (GEA) allows for generation of hypotheses related to the genetic basis of local adaptation by identifying candidate genetic variants associated with variation in environmental gradients, using sequence data from many georeferenced individuals (Lasky et al., 2022). In contrast to gradient forest estimations of genomic offset, which consider alleles as the response variable(s) and environmental variables as predictors, many GEA approaches model the reverse relationship of alleles as predictors of either univariate or multivariate environment, a more natural framework for estimating and interpreting individual locus effects. High ploidy levels and large genomes rife with repetitive elements have historically challenged GEA for many nonmodel invasive grasses, necessitating reduced‐representation approaches that capture only a fraction of full genomic variability. Furthermore, genetic interchange among diverse lineages and species can contribute to bias if DNA sequence from genetically distant ancestors does not align well to the reference genome. Fortunately, alignment‐free approaches for characterizing allele associations with traits are now proving successful for complex plant genomes with significant structural variation (Voichek and Weigel, 2020) and are a promising frontier for GEA. Because they do not require precise estimates of allele copy number, alignment‐free methods are also better suited for analysis of polyploid genomes (VanWallendael and Alvarez, 2022).

As advances in computational methods for integrating high‐dimensional genomic and environmental data sets converge with developments in production‐scale sequencing and alignment‐free bioinformatic approaches, the coming decade offers both substantial challenges and exciting opportunities for botanical informaticists, invasion biologists, and interdisciplinary teams. Embracing the genomic complexity of grasses to estimate their potential for rapid adaptation, wherever they arrive, is even more important as our expanding global economy and rapidly changing climate continue to provide new opportunities for dispersal, establishment, and spread of invasive species.
